# The Preferred Habitat of Reintroduced Banteng (*Bos javanicus*) at the Core and the Edge of Salakphra Wildlife Sanctuary, Thailand

**DOI:** 10.3390/ani13142293

**Published:** 2023-07-13

**Authors:** Rattanawat Chaiyarat, Passorn Ingudomnukul, Nattanicha Yimphrai, Seree Nakbun, Namphung Youngpoy

**Affiliations:** 1 Wildlife and Plant Research Center, Faculty of Environment and Resource Studies, Mahidol University, Nakhon Pathom 73170, Thailandnattanicha.yimpray@icloud.com (N.Y.);; 2 Khao Nam Phu Nature and Wildlife Education Center, Department of National Parks, Wildlife and Plant Conservation, Kanchanaburi 71250, Thailand

**Keywords:** banteng (*Bos javanicus*), area of use, camera trap, Salakphra wildlife sanctuary, species distribution model

## Abstract

**Simple Summary:**

Adaptation to new environments by reintroducing banteng (*Bos javanicus*) is important for sustainable conservation and habitat management. The aims of this study were to test whether increased human disturbance in the edge area affects animal movement, territory size, and even the area of use in Salakphra Wildlife Sanctuary. Using camera traps, we found that the reintroduced banteng preferred salt licks and more distance from ponds. In the core area, the area of use was decreased, followed by the adaptation to the new habitat by the reintroduced banteng. In the edge area, the area of use was smaller than in the core area due to the increased disturbance from human activities. We suggest that the reintroduction program should be conducted in the core area rather than in the edge area, and controlling human activities at the edge may increase the area of use for the reintroduced banteng population in the future.

**Abstract:**

Monitoring of banteng (*Bos javanicus*) after reintroduction is important for their management. This study aimed to monitor the preferred habitat and area of use of reintroduced banteng at the core (13 banteng) and the edge (three banteng) of Salakphra Wildlife Sanctuary between 2019 and 2021 and compared the finding with previous studies conducted from 2014 to 2019. The Binary Logistic Regression (BLR) showed the most preferred, moderately preferred, and least preferred areas were 44.7 km^2^, 1.2 km^2^, and 54.1 km^2^ in the dry season, and 25.9 km^2^, 1.0 km^2^, and 9.3 km^2^ in the wet season, respectively. Maximum Entropy (MaxEnt) showed the most preferred, moderately preferred, and least preferred areas as 12.1 km^2^, 17.3 km^2^, and 65.9 km^2^, respectively. Banteng have previously been found close to ponds and salt licks. The area of use size, as determined by Minimum Convex Polygon (MCP) and Kernel Density Estimation (KDE), was 20.3 km^2^ and 6.5 km^2^, respectively. Three banteng were reintroduced to the edge area in 2020. The edge area was temporarily utilized by these individuals. In the core area, the area of use in this study decreased compared to the previous studies from 2014 to 2019, indicating they were able to find their preferred habitat. This study suggested that, if the area is managed appropriately, banteng will be able to live in a smaller habitat, and we will be able to restore the banteng population in the future.

## 1. Introduction

The study of preferred habitats is important to understand the distribution and abundance of animals [1]. Habitat use refers to the way an animal uses, or consumes, in a generic sense, a collection of physical and biological components (i.e., resources) within a habitat [2]. The basic ecological requirements are important to understand for the monitoring of preferred habitats [3], and adaptation to the new environment [4], of reintroduced species. Species distribution models (SDMs) focus on the role of biotic factors, such as forage quality and quantity [5], as well as other biotic and abiotic factors [6]. Preferred habitats are those that confer the highest fitness, and, thus support the highest equilibrium density, in the absence of other confounding factors [7]. The use of camera trapping for wildlife studies has increased, as it is an efficient, cost-effective, and easily replicable tool to study and monitor terrestrial mammals. Camera trapping is particularly suited to collecting standardized data because sampling can be easily controlled, and the sampling design can be replicated across time and space. The study sites can be contrasted by habitat type, distance to key resources, and vegetation features [8].

The banteng (*Bos javanicus*, family Bovidiae) is one of the famous wild species in southeast Asia and Thailand [9]. Male banteng weigh between 600 and 800 kg and females can weigh between 590 and 670 kg [10]. Their height at the shoulder reaches 1.7 m [10,11]. Banteng are active both during the day and night, but they rest under dense canopies during periods of high temperatures. They prefer open forests and grasslands. A banteng herd is comprised of 10–25 individual females and calves. Males lead solitary lives, only joining the herd during the mating season [12]. Banteng are grazers, with grasses being their main food source, along with visits to salt licks for supplementary nutrients. In the wet season, banteng consumed higher energy forage species (e.g., *Dalbergia cultrate* and *Wrightia arborea*) compared to in the dry season.

In the wild, the forage species of banteng contained higher levels of nitrogen (N) and calcium (Ca) than found in captive forages [13]. Banteng prefer flat plains and being far away from villages [14,15,16,17]. In Indonesia, the most suitable areas for banteng are the secondary forests located in the center portion of the national park. In the dry season, when the main food is scarcer in the forest, the invasive cluster sugar palm provides additional alternative food for banteng. Banteng avoidance behavior is directed more towards dholes (*Cuon alpinus*) than the predator activity areas. The domestic cattle that grazed illegally in the national park appear to be a problem to the banteng since diseases can be contracted from domestic cattle [18]. Siddiq et al. [19] reported that altitude, annual temperature range, distance from the nearest settlement, coastline distance to the settlement, distance to the coastline, altitude, and annual temperature range are the most significant variables for banteng in Meru Betiri National Park, Indonesia. Furthermore, in Sabah, Malaysia, soil associations, distance to intact and logged forests, precipitation in the driest quarter, distance to agroforest and regenerating forest, and distance to oil palm plantations are the environmental factors associated with the distribution of banteng [20]. Nevertheless, Enn et al. [21] reported that salt licks are important as critical temporal resources for banteng in a forest plantation in Sabah, Malaysia.

The horns of banteng are used to decorate homes, and the meat is used for food and local medicine [10]. Unsustainable hunting and habitat loss were the main reasons for dramatic declines and extinction in some areas [10]. The global population has been estimated to be between 4000 and 8000 individuals, primarily located on the mainland of southeast Asia, Malaysia, and Indonesia [9]. In Thailand, the population was estimated at only 500 individuals at the end of the last century [13]. Banteng are considered globally endangered [9], and in Thailand, they are critically endangered [12] and protected under the Reserved and Protected Wildlife Act B.C. 2019.

In Thailand, banteng populations have been decreasing in many areas, but there have been increases in some areas, such as Huai Kha Khaeng Wildlife Sanctuary [22]. In several areas, such as Salakphra Wildlife Sanctuary (SWS), banteng were extinct for over 30 years [16,22], but small populations were kept in zoos and breeding centers [13,16]. Khao Nam Phu Nature and Wildlife Education Center (KNP) is a breeding center that supports ex-situ conservation and provides banteng for the reintroduction program in SWS [16]. In 2014, the first group of banteng (two males and two females) was reintroduced to SWS [17]. As of 2022, 16 banteng (four in 2014, three in 2016, three in 2018, and three in 2019) have been introduced into SWS [23].

This study aimed to monitor the preferred habitat of reintroduced banteng in the core area (undisturbed area) and the edge area (highly disturbed by human activities) to increase the understanding of environmental factors that influence the success of banteng reintroduction in new habitats and to provide insights that can be applied to future reintroductions.

## 2. Materials and Methods

### 2.1. Study Area

**Figure 1:**
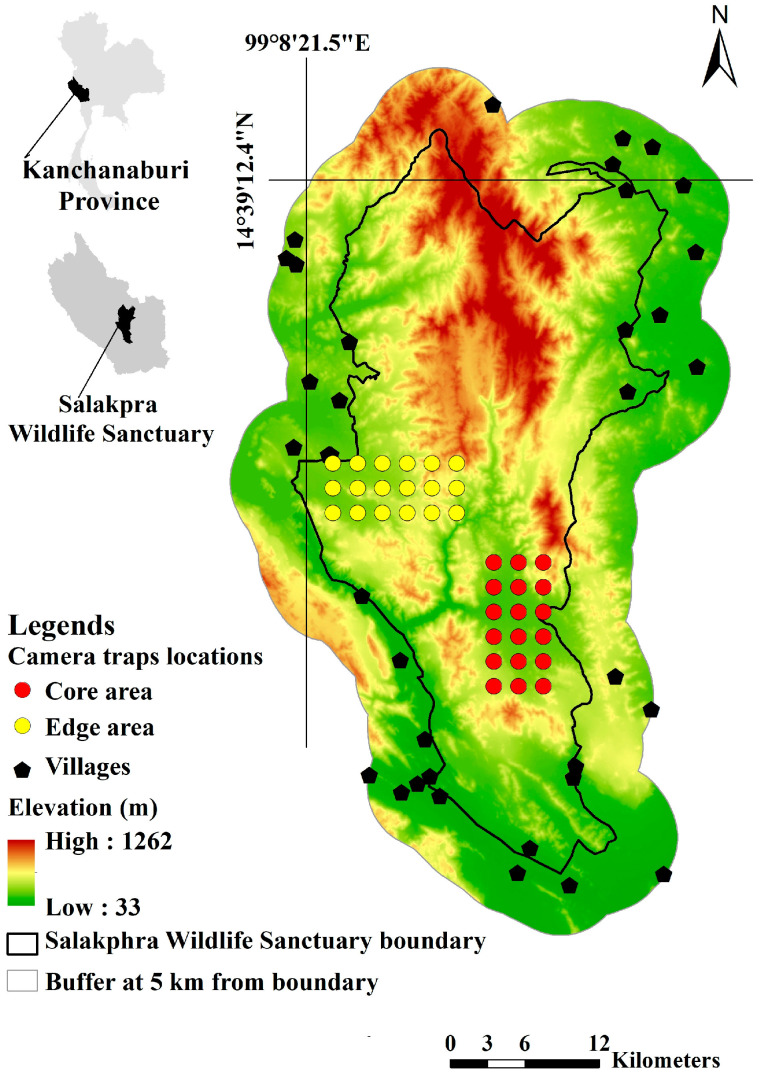
Camera trap locations in Salakphra Wildlife Sanctuary.

Salakphra Wildlife Sanctuary (14°8′37.09″ N, 99°20′33.51″ E, area: ~860 km^2^) is located in Mueang, Bo Phloi, Si Sawat, and Nong Prue district, Kanchanaburi province, Thailand (Figure 1). The altitude of the study site ranges from 700 m to 1000 m above average sea level (asl). The average rainfall is 1071 mm per year and the average temperature is 28 °C. The vegetation cover consists of mixed deciduous forest (60%), dry dipterocarp forest (30%), and disturbed areas (10%). White Crape Myrtle (*Lagerstroemia tomentosa*), Laurel (*Terminalia alata*), Khi-ai, Thai name (*T. triptera*), Myrobalan (*T. bellirica*), and Cambodia Beng Tree (*Afzelia xylocarpa*) are the dominant cover species in the habitat. More than 350 species, including nine wild ungulates, such as gaur (*Bos gaurus*) and samba (*Rusa unicolor*), are found in this area [24]. Salakphra Wildlife Sanctuary was famous for hunting before being established as Thailand’s first wildlife sanctuary in 1965 [12]. The disturbance caused by human activities such as settlements, agriculture practices, tourism, transportation, illegal hunting, wild plant harvesting, and livestock occur more frequently at the edge, rather than in the core area [24] (Table 1).

### 2.2. Data Collection

Data were collected after the reintroduction of 16 banteng as described by Chaiyarat et al. [16,23]. Before reintroduction, the banteng received general medical checkups and minimal human contact, as suggested by Prakobphon [25] and IUCN/SSC [26]. These banteng were trained for six months in the KNP to habituate them to transportation using transportation boxes [16]. Then, the first group (four banteng in 2014), the second group (three banteng in 2016) and the third group (three banteng in 2018) were translocated to a cage used for soft release [23] in the core area, and the fourth group (three banteng in 2019) was translocated to a soft release cage at the edge area of SWS. The animals spent four months in the soft release cage before release. Maize (*Zea mays*), dalgrass (*Hymenachne pseudointerrupta*), Malabar Bindweed (*Hewittia malabarica*), snake gourd (*Trichosanthes cucumerina*), freshwater, and artificial salt licks were provided to the captive-bred banteng. In the training cage, the banteng diets were switched to natural plants found in the area. After reintroduction, natural food plants and salt licks were the main food sources [13,16]. The banteng were monitored every week for a year after being reintroduced [17]. After a year of reintroduction, when the radio signals were no longer available, the camera traps were employed to monitor the preferred habitat preferred of these reintroduced banteng.

Cameras traps (Bushnell 12 MP Trophy Cam HD Essential Trail Camera, Suresnes, France) were installed between 2019 and 2021. These camera traps were used to collect data to perform SDMs, study the preferred habitat, and estimate the area of use of the 16 banteng. Thirteen banteng were reintroduced to the core area, and three others were reintroduced to the edge area. These data were compared to the study species distribution models and preferred habitats and were used to monitor seven reintroduced banteng in the core area between 2014 and 2019, as described by Chaiyarat et al. [17]. Memory cards and batteries were changed every month at each location for the entire two years of the study period. Camera trap locations were selected based on the banteng locations that were detected using a radio transmitter [27,28]. Therefore, water sources [29], salt licks, and wildlife trails [30], which were often visited by banteng and other large mammals, such as wild Asian elephants (*Elephas maximus*) and gaur and sambar deer, at the selected camera trap locations, were primarily used as locations for camera trap placement [17,29].

Each camera trap location was installed about 0.75 m off the ground [31], with two cameras opposite to each other, positioned to photograph both of the asymmetrical flanks of the banteng for positive identification [32]. Camera traps were installed in 18 (2 × 2 km^2^) places per study area and operated continuously, 24 h per day. The pictures had a resolution of 1648 × 1236 pixels (Figure 1). Camera ID, time, date, and temperature were also recorded for each exposure and were stamped on the photographs [17] (Figure 1).

### 2.3. Data Analysis

#### 2.3.1. Species Distribution Models and Preferred Habitat

Binary Logistic Regression (BLR) [33] and Maximum Entropy (MaxEnt) software for modeling species niches and distributions, version 3.4.1 [34,35,36], were used to evaluate the species distribution model and preferred habitat. BLR was used and fitted to study the topographic attributes associated with the presence and absence [37] of banteng, and other wildlife species, such as elephants [38]. Presence is a probabilistic function mainly affected by species abundance and detectability. The camera trap locations captured banteng photographs, which were recorded and imported onto a digital map [23]. The absences, in the present study, were the locations of camera traps that were not able to capture banteng photographs. However, the assumption that absence indicates areas where banteng were not present because of a negative species and environment relationship is not necessarily a valid interpretation [39,40]. Habitat composition was analyzed from the land use layers of the SWS digital map [24]. Topography data were obtained from a digital elevation model (DEM) generated by SWS [24] from 1:50,000 topographic maps. Elevation was used to generate the slope and was then resampled to a 30 m pixels resolution. The distance between each banteng observation point and the environmental parameters was estimated [23].

The Species Distribution Models (SDMs) focused on the role of biotic and abiotic factors [6], such as land use types (lu62), which were used as food sources and cover; aspect and elevation (elev, m), which was important for vegetation cover; distance to salt lick (lick, km), which was a mineral supplement provided for most herbivore species; distance to artificial pond (pond, km), which was a water source; distance to forest road (road, km), which affected to the distribution; slope (%), which was important for distribution; distance to the wildlife sanctuary guard station (station), which was a safe zone from predators and poachers; distance to a natural stream (stream), which was the water source and distance to village (village), which affected the distribution. Preferred habitats were those that conferred the highest fitness, and, thus support the highest equilibrium density in the absence of other confounding factors [8]. The habitat parameters were imported into the R program [37] for BLR analysis and model-fit statistics. In general, model precision was greater when models were developed from data that included more sites where the study animal was absent than it is with data that included more sites where they were present, perhaps because most of the study sites, on average, contained more non-preferred than preferred habitats [40].

Maximum entropy (MaxEnt) creates better models from small sample sizes compared to other modeling methods [41,42,43,44]. The MaxEnt uses presence-only data to predict the distribution of a species [36]. The estimation of species distribution probability is most accurate when the species occurrence is closest to the uniform environmental limitation [43]. In this study, the four habitat categories were divided into highly (>75–100), moderately (>50–75), low (>25–50), and non-preferred habitats (<25).

Species Distribution Models are usually evaluated by preferred significance with cross-validation. The area under the ROC curve (AUC) value is the most commonly used statistic to evaluate SDMs results and is classically used with climatic variables that were strongly interrelated with each other. In general, the AUC ranges from 0 to 1, where a score of 1 indicates perfect discrimination, a score of 0.5 implies predictive discrimination that is no better than a random guess, and values <0.5 indicate performance worse than random [44,45]. There is a strong relationship between the AUC and sensitivity (the proportion of correctly predicted presence locations), which is equivalent to specificity (the proportion of correctly predicted absence locations) [46].

#### 2.3.2. Model Performance

The replicates were tested and the omission rate and predicted areas as a function of the cumulative threshold, averaged over replicated runs, were computed to predict the SDM performance. The receiver operating characteristic (ROC) curve was produced with the same data, once again averaged over the replicated runs, and received lower, median, minimum, maximum, average, and standard deviation from all runs [47]. Further, the jackknife procedure and percentage variable contributions were used to estimate the relative influence of different predictor variables. The AUC was used to evaluate model performance [48,49].

#### 2.3.3. Estimating the Area of Use by Reintroduced Banteng

The minimum convex polygon (MCP) and the kernel density estimation (KDE) bounds on the innermost 95% of the density of the presence data points in the dry and wet seasons were used to estimate the size of the area of use [50] by the reintroduced banteng. The model derived from this equation was used to create an area-of-use map in ArcGIS (10.4.1) [51]. This area of use was based on the camera trap locations that covered all signal locations of banteng in the first year of reintroduction.

The MCP was used to determine the home ranges of the banteng population after reintroduction. The interpretation and comparison of the size of the area of use by the reintroduced banteng were assessed using the 95% MCP. The use of MCP was justified because of the sample size and the temporally clustered nature of fixes that resulted in the autocorrelation of results [16,17,52]. The MCP is one of the oldest techniques for home-range estimation, and it is globally comparable among species, and its inclusion as one or more methods of range calculation is, therefore, valuable [50]. The MPCs were generated in ArcGIS 10.4.1 [51] to estimate the area of use size of the reintroduced banteng. The size of the area of use by the reintroduced banteng was estimated in different seasons. In the dry season, there were two camera trap locations at the edge and nine camera trap locations in the core area, while in the wet season, there were three camera trap locations at the edge. The KDE bounds of the innermost 95% of the presence data points were used to estimate the size and created the map of the area of use [53] of the reintroduced banteng.

## 3. Results

### 3.1. Species Distribution Models and Preferred Habitat

**Figure 2:**
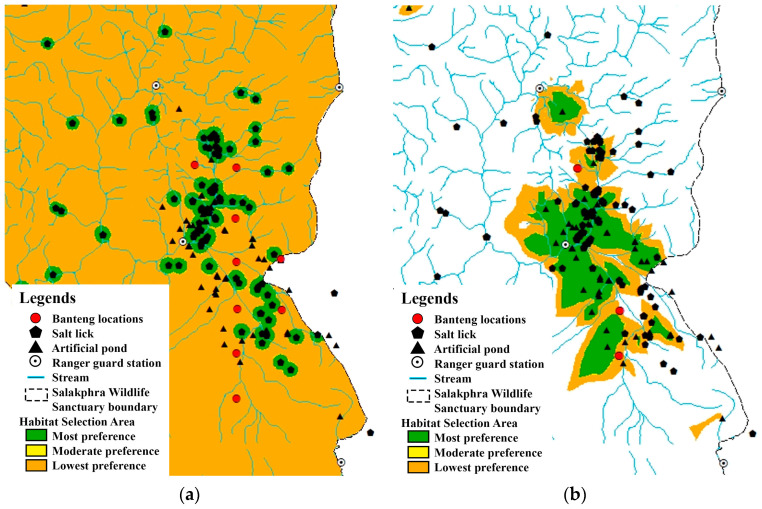
Location of camera traps that captured the reintroduced banteng: (**a**) the dry season and the whole year and (**b**) the wet season at the core area of Salakphra Wildlife Sanctuary, Thailand.

The BLR was used to calculate the habitat model of the reintroduced banteng in the core and edge areas of SWS. In each area, 18 camera trap locations were used to monitor the presence and absence of banteng, considering each environmental factor. In the core area, the camera traps captured photos of banteng in nine locations in the dry season and three locations in the wet season. The results of the analysis of the BLR models are shown in Table 2 and Figure 2. For the dry season and the whole year, the coefficient of the BLR showed that the preferred habitat of the banteng had a high distance from streams and was close to the wildlife sanctuary guard stations and artificial ponds. In the wet season, the banteng were found to be further from the salt licks and agricultural areas and stayed at lower elevations.

**Figure 3:**
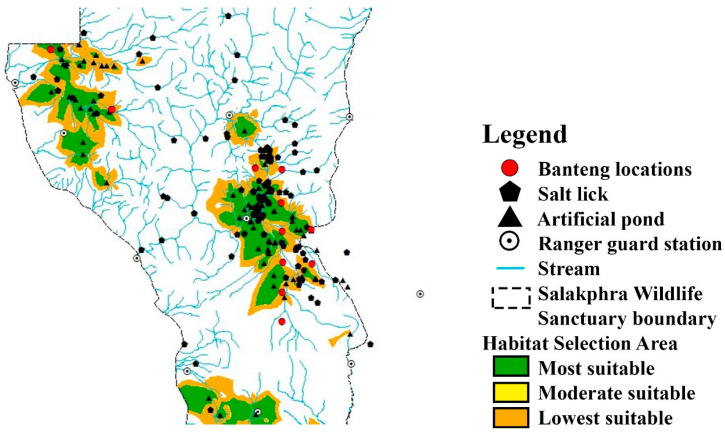
Location of camera traps that captured the reintroduced banteng at an edge area of Salakphra Wildlife Sanctuary in the dry season and the whole year.

At the edge area, banteng were found in two out of 18 camera trap locations in the dry season. This area was a temporary habitat for reintroduced banteng. In the dry season, the coefficient of the BLR showed that the area of use by the banteng was far from the temporary streams, but close to permanent artificial ponds (Table 1 and Figure 3).

**Figure 4:**
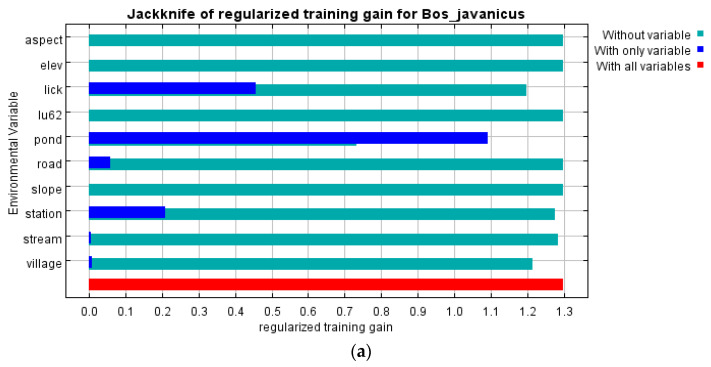

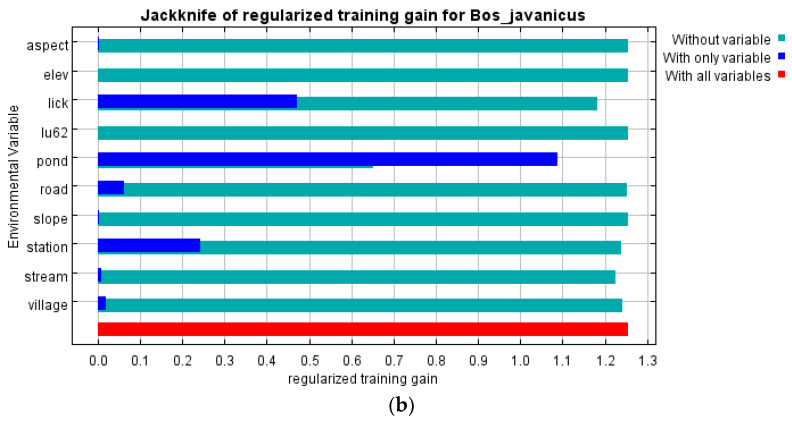
The receiver-operating characteristic (ROC) curve plot with evenly spaced thresholds marked along the ROC curves and area under the ROC (AUC = 0.904 in both the core and edge areas), calculated by MaxEnt, and the jackknife test for contributions of the variables of the reintroduced banteng habitat models (**a**) in the core area and (**b**) in the edge area of Salakphra Wildlife Sanctuary, Thailand. Jackknife test of regularized training gain. Dark blue columns show how the model gain would be using each variable in isolation. Light blue columns show the change in the model gain if the variable was excluded. The longest dark blue column indicates the variable with the most useful information by itself. The shortest light blue column indicates the variable which has the most information that was not present in other variables. Aspect; elevation was given in m; distance from salt lick (m); lu2562 = land use types in 2019; distance from artificial pond (m); distance from forest road (m); slope (%); distance from Salakphra Wildlife Sanctuary Guard Station (m); distance from stream (m) and distance from village (m).

MaxEnt was used to calculate the habitat model of the reintroduced banteng in the core and edge areas of SWS. The results showed that all 15 generated training or testing models (for all records modeled) had a high level of performance compared with a random model (AUC = 0.904). The contribution of the environmental variables and the results of the jackknife-test analysis are given in Figure 4.

In the dry season, the BLR analysis showed that the areas were highly preferred by banteng. The total preferred habitat (highly, moderate, and less preferred) of reintroduced banteng in the dry season was 2.8 times greater than in the wet season. In the dry season, banteng utilized the habitats close to streams, but in the wet season, they utilized areas close to salt licks and avoided the areas close to human activities. MaxEnt showed that the combined highly and moderately preferred area was 29.4 km^2^. Most of the habitat in the core area was less preferred. At the edge, BLR and MaxEnt showed that banteng selected areas close to artificial ponds and salt licks.

**Figure 5:**
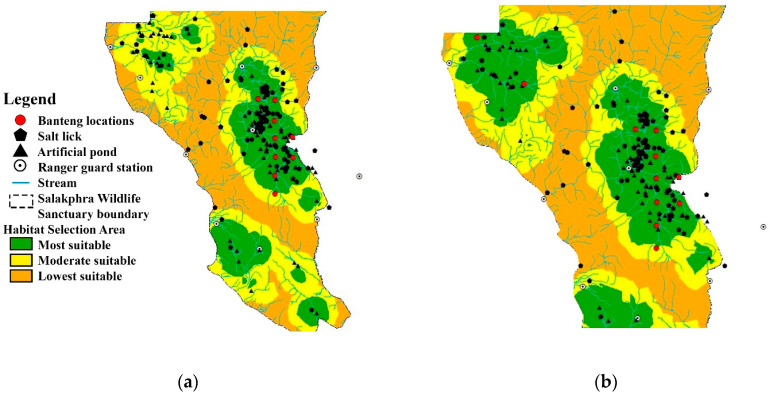
Location of camera traps that captured the photographs of reintroduced banteng by used MaxEnt: (**a**) at the core area and (**b**) at the edge of Salakphra Wildlife Sanctuary, Thailand.

MaxEnt showed that the core area was the most preferred for banteng. This area was flat and close to water and salt licks (regularized training gain > 0.4). The percent contribution was 82.8 and 12.6 (AUC = 0.904), respectively (Figure 5). The preferred area measured 15.7 km^2^; while the moderately preferred area measured 15.7 km^2^, and the least preferred area measured 64 km^2^ (Table 3).

MaxEnt showed that the core area of SWS was the most preferred habitat for banteng. The environmental factors with the highest percent contribution associated with the distribution of banteng were distance to artificial ponds and salt licks (regularized training gain > 0.4) at 80.7% and 12.8%, respectively (AUC = 0.904). The high, moderate, and low preferred areas were 12.1 km^2^, 17.3 km^2^, and 65.9 km^2^, respectively (Figure 4 and Figure 5, and Table 3). At the edge area, the high, moderate, and less preferred areas were 44.7 km^2^, 1.2 km^2^, and 54.1 km^2^, respectively. The environmental factors with the greatest percent contribution associated with the distribution of banteng were distance to streams, artificial ponds, and wildlife sanctuary guard stations (Figure 4 and Figure 5, and Table 3).

### 3.2. The Size of Area of Use by Reintroduced Banteng

**Figure 6:**
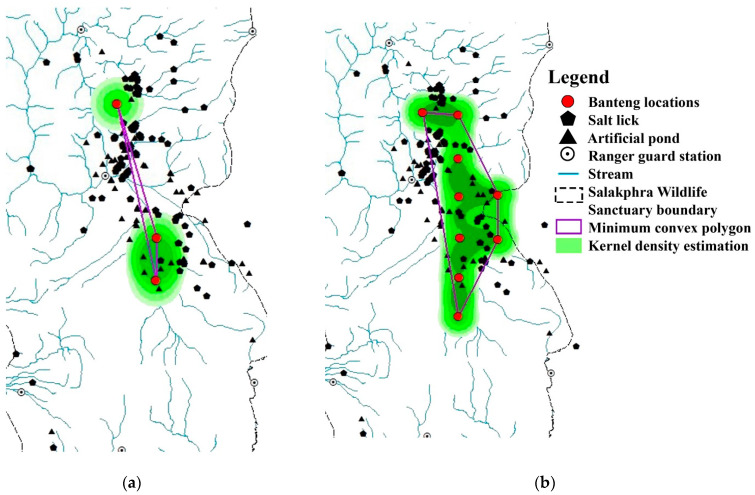
Location of camera traps that captured photographs of reintroduced banteng: (**a**) in the dry season and (**b**) in the wet season in the core area of Salakphra Wildlife Sanctuary, Thailand. The 1.9 km^2^ area resulted from the small sample size and the poor configuration of the three points.

Calculations based on fixed camera trap locations using the 95% MCP and KDE found that the size of the potential area of use, by employing 95% MCP of reintroduced banteng, was smaller in the wet season (20.3 km^2^) than in the dry season (1.9 km^2^). Furthermore, 95% of KDE was larger in the dry season (7.5 km^2^) than in the wet season (6.5 km^2^) (Table 3 and Figure 6).

Between the study performed in 2014–2019 conducted by Chaiyarat et al. [17] and the current study considering 2019–2021, the preferred habitat preferred area and the size of the area of use by reintroduced banteng have decreased. In the dry season, the BLR model for 2019–2021, was smaller than that of 2014–2019 [17] in all preferred habitat categories; highly preferred habitat decreased by 95.3% (44.7 km^2^), moderately decreased by 89.8% (1.2 km^2^), and the less preferred habitat decreased by 94.6% (54.1 km^2^). In the wet season, the BLR model of highly preferred habitat was higher in 2019–2021 (+65.6%, 25.9 km^2^), while the moderately preferred habitat decreased by 99.1% (1 km^2^), and the low preferred habitat decreased by 98.9% (9.3 km^2^) (Table 3). In 2019–2021, the MaxEnt model showed that all the preferred habitats decreased compared to 2014–2019; highly preferred decreased by 11.7% (12.1 km^2^), moderately preferred decreased by 48.5% (17.3 km^2^), and the less preferred habitat decreased by 96.5% (65.9 km^2^) (Table 3).

The area calculated as 95% of MCP decreased in the dry season by 90.3% (1.9 km^2^), while in the wet season, it increased by +3.9 (20.3 km^2^), and increased by +15.8 (20.3 km^2^) for the whole year (Table 3). On the other hand, the 95% of KDE in the dry season increased by 21.3% (7.5 km^2^) in the dry season, decreased by 61.1% (6.5 km^2^) in the wet season, and decreased by 64.1% (6.5 km^2^) for the whole year (Table 3).

## 4. Discussion

### 4.1. Species Distribution Models and Preferred Habitat

The reintroduced banteng in SWS adapted well to the food, plants, and environment after reintroduction [13]. Their diet was broadly similar to that of introduced banteng in Khao Khieo-Khao Chomphu Wildlife Sanctuary (KKKC) [16], and those in northern Australia [54], and the native banteng in Huai Kha Kaeng Wildlife Sanctuary (HKK) [55]. The habitat model of the reintroduced banteng using BLR and MaxEnt had a very high level of performance compared to a random model, where the AUC was over 0.9 and close to the training AUC values [56,57]. These two models were suitable for the study of the preferred habitat of reintroduced banteng. The contribution of environmental variables and the results of the jackknife-test analysis of MaxEnt showed that the distance from the ponds contained more useful information. This finding was different from previously reported results [17], which indicated that low distance from salt licks, as they are rich in minerals, or feeding sites, which are important for critical temporal use for banteng [21], and the long distance from villages, as they disturb the distribution of banteng [19], were that the most important environment variables. The next most important variables were elevation, as banteng prefer the lowlands and open forest [19], land use, and distance from the SWS Guard Station, due to a lower risk of poaching and predators [18].

In the dry season, the BLR showed that the core area was highly preferred by banteng. The total preferred habitat (highly, moderately, and less preferred) was 2.8 times greater in the dry season than in the wet season. In the dry season, the forage species and the water sources were decreased and scattered [17,29], causing the banteng to expand their range due to the limited resources. As a result, the need to walk a greater distance increased the size of the area of use of the reintroduced banteng, as suggested by the food limitation hypothesis [58]. Consequently, the preferred habitat area for banteng decreased in the dry season. In the lowland forest of Indonesia, the presence of the invasive cluster sugar palm (*Arenga obtusifolia*) in the dry season provides additional alternative food for banteng when its main food is scarcer in the forest [18]. They tend to live close to streams due to other water sources drying up. In the wet season, banteng can find water sources almost everywhere and they live close to salt licks, where the soil is wet and soft [13,14,15,59]. In Sabah, Malaysia, Lim et al. [20] found that the soils were the suitable environmental factors associated with the distribution of banteng. Furthermore, Chaiyarat et al. [13] found that the quality of food plants in SWS is low. This may be the reason why the habitat areas used by banteng were larger in the dry season than the wet season, as found in semi-free-range cattle [60] that have the same biological characteristics, and in translocated roe deer (*Capareolus capreolus*) in Mediterranean habitats [61], which exhibit similar patterns. Fecal analysis of accidentally introduced banteng in KKKC [16] and in northern Australia [52] confirmed that some dietary items, especially grass species, may be limited in the dry season [62]. This forces banteng to change their forage species to dicotyledons, as found in reintroduced bison (*Bison bison*) in the USA [63].

The BLR and MaxEnt models showed the preferred habitat of banateng was located in the core area of SWS, where flat plains are located [17], similar to finding in KKKC [16] and HKK [59], and also due to its close distance to water sources and salt licks [15,16,17,59]. MaxEnt showed a higher preferred habitat area for banteng in the core area of SWS compared to the edge area. The environmental factors with the greatest percent contribution to the distribution of banteng were the distance to artificial ponds and salt licks, as these were used to supplement the minerals in the food plants [13].

The MPC and KDE analyses showed a different trend. In the dry season, the 95% MPC was lower than in the wet season, while the 95% KDE was higher than in the wet season. The reason for this was the banteng photographs were captured in a few camera trap locations in the dry season, but this was not sufficient to estimate the real MCP and must be evaluated with care. During the dry season, banteng may have used much larger ranges than in the wet season [59] and moved outside of the camera trap locations. This was supported by the results from BLR and MaxEnt. Although the area of use size of reintroduced banteng was smaller than found in previous studies [17], this can be explained by their adaptation to the new habitat and the time required to search for the preferred habitat being reduced [64]. Furthermore, there seemed to be differences between the core and the edge areas; there were more human disturbances in the edge area, which can affect the reintroduced banteng movement, territory size, and even area of use, as found in the native banteng of Mae Wong National Park, Thailand, which were recovered after local extinction over 30 years [22].

In the core area, the preferred habitat area and the size of the area of use by reintroduced banteng in this study (2019–2021) decreased compared to the period of 2014–2019 [17]. This indicated that the reintroduced banteng could adapt to the new habitats and find their preferred habitat for survival. Usually, the preferred habitat of the reintroduced wildlife reduces after they were adapted to the new habitat [26]. After the reintroduction of banteng in the core area of SWS, the body condition scores of all individuals increased in the first year, especially in the males [14], leading to large body size, rapid bulking up, and expression of more aggression, especially during the mating period [65]. Conversely, the steady improvement of the females’ body condition may have been to parturition and lactation during their maturity period, similar to dairy cows [66] and elephants [29,67]. At the edge area, despite the presence of mixed deciduous forest, which is preferable for banteng [16], high levels of disturbance from human activities and competition with the domestic cattle were influenced the preferred habitat of the reintroduced banteng, as found in KKKC, where some agricultural areas were present in the habitat, making it less suitable overall [55].

### 4.2. Recommendations for Banteng Conservation and Habitat Management

The key factors that influence conservation of reintroduced banteng are habitat management and genetic conservation [68]. The fluctuations in preferred habitat can reflect the quality and quantity of resources and other factors such as parasites or even mortality [68]. Improving grazing quality in the agricultural areas inside the wildlife sanctuary and enlarging the wildlife sanctuary into surrounding cropland might help increase the population. The edge area between the border of SWS and adjacent agricultural areas might further promote human and banteng conflict and poaching [69] and increase the risk of disease transmission from domestic livestock [16]. Lack of knowledge and application is an indirect threat to the long-term conservation of the banteng population, as in the case of the Borneon elephant [70].

Improving public awareness by conducting outreach programs and strict law enforcement by patrolling, combined with habitat management measures, along the habitat site, might reduce human and banteng conflict in the area [16]. This research might be used to improve knowledge of the preferred habitat, which will have relevance for future planned translocations.

### 4.3. The Limitations of the Study

Our study had several limitations. Most importantly, inferences were based on a small number of reintroduced banteng (*n* = 13 at the core and three at the edge) and a small number of camera trap locations (*n* = 18 in each area). Nonetheless, as bantengs live in a group and only mature males are solitary, but still follow the herd and share the common resources with them. Furthermore, after the second year, the radio-collars were not available, necessitating the camera trap method suitable to monitor the reintroduced banteng in this area. A larger sample size would provide greater confidence in our conclusions about the preferred habitat. Unfortunately, the limitation of camera trap locations may have affected our understanding of the preferred habitat and the size of the area of use of the reintroduced banteng. In this case, before installing the camera trap, the researchers must be confident that the camera trap locations cover the distribution range of the herd. For future studies, increasing the number of camera trap locations and reducing the distance between each camera trap is recommended.

## 5. Conclusions

In conclusion, our study provides new information on the preferred habitat of banteng in two different areas. The results showed that the reintroduced banteng preferred the core habitat area rather than the edge habitat area of SWS due to the poor conditions of the edge area, such as lower availability of food plants, human disturbance, and other environmental factors. Our predictive preferred habitat maps between the core and the edge habitat sites can serve as an important first approximation of areas of conservation interest for the endangered banteng. These maps should be considered in land use planning and environmental assessment processes within their range. The areas considered for the reintroduction of new populations or expansion of existing populations should prioritize identifying and protecting these key habitats to support banteng population growth and recovery.

## Figures and Tables

**Table 1:** Comparisons of environmental factors between core and edge habitats of Salakphra Wildlife Sanctuary.

Environmental Factors	Edge Habitat	Core Habitat
Land use types (lu62)	Mixed deciduous	Mixed deciduous
Aspect	Flat	Flat
Elevation (elev, m)	Low	High
Distance to salt lick (lick, km)	Far	Near
Distance to artificial pond (pond, km)	Far	Near
Distance to forest road (road, km)	Near	Far
Slope (%)	Low	Low
Distance to wildlife sanctuary guard station (station)	Far	Near
Distance to natural stream (stream)	Far	Near
Distance to village (village)	Near	Far

**Table 2:** Binary logistic regression models predicting the habitat reference of banteng after reintroduction in the core area of Salakphra Wildlife Sanctuary in the dry season and the wet season by camera trap detection. The Akaike information criterion (AICs) was used to select the preferred habitat model.

Model			Dry Season				Wet Season				
	AICs	Factor	Estimation	S.E.	z	Pr(>|z|)	Factor	Estimation	S.E.	z	Pr(>|z|)
Core											
Step 1	20	Intercept	−14.3	2.5 × 10^6^	0	1	Intercept	−0.7	1.1 × 10^6^	0	1
		Road	−177	1.1 × 10^7^	0	1	Road	−6.5	7.8 × 10^5^	0	1
		Stream	23.5	1.8 × 10^6^	0	1	Stream	−2.5	5.7 × 10^5^	0	1
		Station	25.7	4.2 × 10^5^	0	1	Station	26.7	2.1 × 10^5^	0	1
		Salt lick	−64.5	3.6 × 10^6^	0	1	Salt lick	41.8	1.6 × 10^6^	0	1
		Agriculture	−48.8	2.5 × 10^6^	0	1	Agriculture	24.6	8.2 × 10^5^	0	1
		Plantation	135	1.2 × 10^7^	0	1	Plantation	20.2	2.1 × 10^6^	0	1
		Village	57.5	2.9 × 10^6^	0	1	Village	8.8	1.6 × 10^6^	0	1
		Elevation	−0.3	3.1 × 10^6^	0	1	Elevation	−52.3	4.6 × 10^5^	0	1
		Slope	−179	9.5 × 10^6^	0	1	Slope	−26.1	3.4 × 10^6^	0	1
Step 2	8	Intercept	115	7.8 × 10^4^	0.001	0.999	Intercept	21.4	4.2 × 10^4^	0.001	0.999
		Stream	0.2	132	0.002	0.999	Salt lick	253.5	2.2 × 10^5^	0.001	0.999
		Station	−0.02	24.2	−0.001	0.999	Agriculture	149.4	1.3 × 10^5^	0.001	0.999
		Artificial pond	−0.2	91.4	−0.002	0.999	Elevation	−269.3	2.3 × 10^5^	−0.001	0.999
Edge											
Step 1	20	Intercept	−14.3	2.5 × 10^6^	0	1	N/A	N/A	N/A	N/A	N/A
		Road	−177	1.1 10^7^	0	1	N/A	N/A	N/A	N/A	N/A
		Stream	23.5	1.8 × 10^6^	0	1	N/A	N/A	N/A	N/A	N/A
		Station	25.7	4.2 × 10^5^	0	1	N/A	N/A	N/A	N/A	N/A
		Salt lick	−64.5	3.6 × 10^6^	0	1	N/A	N/A	N/A	N/A	N/A
		Agriculture	−48.8	2.5 × 10^6^	0	1	N/A	N/A	N/A	N/A	N/A
		Plantation	135	1.2 × 10^7^	0	1	N/A	N/A	N/A	N/A	N/A
		Village	57.5	2.9 × 10^6^	0	1	N/A	N/A	N/A	N/A	N/A
		Elevation	57.5	3.1 × 10^6^	0	1	N/A	N/A	N/A	N/A	N/A
		Slope	−179	9.5 × 10^6^	0	1	N/A	N/A	N/A	N/A	N/A
Step 2	8	Intercept	21.4	7.8 × 10^5^	0.001	0.999	N/A	N/A	N/A	N/A	N/A
		Salt lick	253.5	132	0.002	0.999	N/A	N/A	N/A	N/A	N/A
		Agriculture	149.4	24.2	−0.001	0.999	N/A	N/A	N/A	N/A	N/A
		Elevation	−269.3	91.4	−0.002	0.999	N/A	N/A	N/A	N/A	N/A

N/A = the number of present points was lower than the minimum points of the program required for analysis. The present points in the dry season were equal to the whole year.

**Table 3:** Binary logistic regression models (BLR), maximum entropy (MaxEnt), minimum convex polygon and Kernel density estimation (KDE) predicting habitat suitability of banteng after reintroduction in the core area of Salakphra Wildlife Sanctuary in the dry season, the wet season, and the whole year using camera trap detection between (a) 2014 and 2019 and (b) 2019 and 2021.

Model	Preferred Area (km^2^)	(a) 2014–2019 ^†^			(b) 2019–2021 ^‡^			Difference between (a) and (b) (%)		
		Whole	Dry	Wet	Whole	Dry	Wet	Whole	Dry	Wet
BLR	High	15.4	956.8	8.9	25.9	44.7	25.9	+40.5	−95.3	+65.6
	Moderate	36.7	11.8	120.7	1.0	1.21	1.0	−97.2	−89.7	−99.1
	Low	918.6	2.9	835.9	9.3	54.1	9.3	−99.0	+94.6	−98.9
MaxEnt	High	13.7	12.2	4.9	12.1	N/A	N/A	−11.5	N/A	N/A
	Moderate	33.6	40.3	22.2	17.3	N/A	N/A	−48.5	N/A	N/A
	Low	1878.4	1873.2	1898.6	65.9	N/A	N/A	−96.5	N/A	N/A
MCP (%)	95	17.1	19.5	19.5	20.3	1.9	20.3	+15.8	−90.3	+3.9
KDE (%)	100	18.2	5.9	16.8	18.7	18.9	18.7	−2.7	+68.8	+10.2
	95	18.1	5.9	16.7	6.5	7.5	6.5	−64.1	+21.3	−61.1
	80	3.9	5.9	4.3	5.5	5.8	5.5	+29.1	−1.7	+21.8
	60	3.1	4.0	4.4	4.9	4.3	4.9	+36.7	+7.0	+10.2
	40	2.4	4.0	2.6	5.8	2.9	5.8	+58.6	−27.5	+55.2
	20	1.4	1.4	1.8	8.6	1.8	8.6	+83.7	+22.2	+79.1
	10	0.9	0.4	0.6	0.4	0.6	0.4	−55.6	+33.3	−33.3

^†^ Adapted with permission from Chaiyarat et al. [17], 2019, Rattanawat Chaiyarat; ^‡^ Data from this study; − was decreased; + was increased.

## Data Availability

Not applicable.
